# Circulating immunoglobulins and transient lymphocytopenia in a sub-study of CAPRISA 012B, testing HIV monoclonal antibodies in a phase 1 trial

**DOI:** 10.1038/s41598-024-63902-2

**Published:** 2024-06-12

**Authors:** Parveen Sobia, Sharana Mahomed, Aida Sivro, Stephane Paul, Farzana Osman, Ishana Harkoo, Nigel Garrett, Quarraisha Abdool Karim, Salim S. Abdool Karim, Derseree Archary

**Affiliations:** 1 Centre for the AIDS Programme of Research in South Africa (CAPRISA), University of KwaZulu-Natal, 2nd Floor, Doris Duke Medical Research Institute, 719 Umbilo Road, Durban, 4041 South Africa; 2 Department of Medical Microbiology, University of Kwazulu-Natal, Durban, South Africa; 3 JC Wilt Infectious Disease Research Centre, National Microbiology Laboratory, Public Health Agency of Canada, Winnipeg, MB Canada; 4 Department of Medical Microbiology and Infectious Diseases, University of Manitoba, Winnipeg, MB Canada; 5 Department of Epidemiology, Columbia University, New York, NY USA; 6 GIMAP (EA3064), University of Saint-Etienne/University of Lyon, Saint-Étienne, France

**Keywords:** Immunology, Diseases, Health care

## Abstract

Acute, transient lymphocytopenia, not clinically significant was observed in the CAPRISA 012B phase 1 clinical trial following administration of broadly neutralizing antibodies (bnAb)-CAP256V2LS alone or with VRC07-523LS. Lymphocytopenia was assigned upon a > 50% decline in absolute lymphocyte counts following bnAb administration. We posited that systemic immunoglobulins (Igs), and cytokine profiles of eight women who developed lymphocytopenia were different to the 12 women without lymphocytopenia. Plasma Ig subclasses (IgG)/isotypes (IgM/IgA), and 27 cytokines were measured at enrolment (prior to bnAbs) and at days 1, 7, 28, 56 post-bnAb administration. IgG subclasses, IgM and total lymphocyte counts were significantly lower prior to bnAbs in women with gradable lymphocytopenia than those without. Gradable lymphocytopenia compared to non-lymphocytopenia women had significantly higher MIP-1β from enrolment up to day 56. TNF-α was significantly lower in gradable lymphocytopenia compared to non-lymphocytopenia women for enrolment, days 7, 28 and 56 except for day 1. Within the gradable and within the non-lymphocytopenia women, from enrolment to day 1, significantly elevated IL-6, IL-8, IP-10, MCP-1, G-CSF and IL-1RA were found. Additionally, within the gradable lymphocytopenia women, 9 additional cytokines (TNF-α, MIP-1α, MIP-1β, RANTES, Basic FGF, eotaxin, IFN-γ, IL-17A and IL-4) were significantly elevated at day 1 post-bnAbs compared to enrolment. This sub study presents preliminary findings to support the monitoring of baseline immunological markers including lymphocyte counts for assessing the development of transient lymphocytopenia. In high-risk settings conducting clinical trials testing bnAbs for HIV prevention, understanding factors that could amplify rates of lymphocytopenia, even if transient, remain undefined.

## Introduction

High incidence of HIV infections among young women in sub-Saharan Africa remains a major concern^1,2^, however the development of an effective vaccine remains a challenge.

Clinical trials assessing passive immunization of potent broadly neutralizing antibodies (bnAbs) for an HIV prevention indication are currently underway. Recent findings from the Antibody-Mediated Prevention (AMP) efficacy trials demonstrated that breakthrough HIV infections with viruses sensitive to neutralization by VRC01 were 75% lower compared to the placebo group. These findings support the further assessment of bnAbs for HIV prevention^3^. The Centre for the AIDS Programme of Research in South Africa (CAPRISA) is currently evaluating three bnAbs (VRC07-523LS, PGT121 and CAP256V2LS) in a suite of trials^4^. CAPRISA 012B is a first-in-human phase 1 dose-escalation study of the safety, tolerability, and pharmacokinetics of the bnAb CAP256V2LS alone and in combination with VRC07-523LS, conducted in Durban, South Africa^4^.

The native antibody, CAP256-VRC26.25 was isolated from a clade C virus-infected person living with HIV (PLWH) in the CAPRISA 002 Acute Infection study^5^. CAP256-VRC26.25 targets the V2 region of the HIV-1 envelope and was found to be highly potent^6^. The improved LS version of CAP256-VRC26.25 was engineered to increase the plasma half-life, prevent proteolytic cleavage, and improve production^7^. The final variant of the antibody-CAP256V2LS retained its original potency and breadth and was used in the clinical trial.

In clinical trials assessing monoclonal antibodies for cancer therapies, peripheral blood lymphocytes are frequently evaluated and clinically significant lymphocytopenia has been reported^8–10^. In the context of oncology, the development of lymphocytopenia is an important finding as it modifies the risk for other opportunistic infections^11,12^.

Previous studies have shown that patients with IgG deficiency have significantly lower T and B cell counts^13^. Other studies have demonstrated an upregulation of cytokines in patients with lymphocytopenia^14,15^. Although the mechanism remains unclear, the synergistic action of different cytokines, chemokines and growth factors can trigger lymphocytopenia. In addition, deficiency of immunoglobulins (Igs) can be attributed to cytokine dysregulation. Cytokines also regulate the IgG subclass and isotype switching which can then alter the profile of Igs^16^. Therefore, characterizing the Igs and cytokines provides important information about putative immune-mediated mechanisms in the genesis of lymphocytopenia including T cell margination and/or tissue re-distribution.

During the conduct of the CAPRISA 012B trial, a subset of women experienced acute lymphocytopenia post CAP256V2LS-administration alone or in combination with VRC07-523LS. We aimed to characterize the circulating immunoglobulin (Ig) isotypes, IgG subclasses, and cytokine responses of eight women who experienced lymphocytopenia. We hypothesized that women who developed gradable lymphocytopenia exhibited different levels of certain pro-inflammatory cytokines and immunoglobulin subclasses and isotypes compared to those who did not. These immunological markers were measured prior to any administration of bnAbs and after. To our knowledge, this is the first study that describes the various IgG subclasses, isotypes and cytokine responses in African women who developed acute, transient lymphocytopenia in a monoclonal antibody trial for HIV prevention.

## Methods

### Study participants and sample collection

In CAPRISA 012B, CAP256-V2LS was administered intravenously to HIV uninfected and infected healthy women or subcutaneously alone and in combination with VRC07523LS to HIV uninfected women in the presence or absence of a dispersing agent which is a recombinant human hyaluronidase called ENHANZE® drug product (EDP)^4^. Twenty participants from this trial were included in this study. The trial was approved by the South African Health Products Regulatory Authority (SAHPRA) and the University of KwaZulu-Natal Biomedical Research Ethics Committee (BREC) (trial reference numbers: BREC00000857/2019 and SAHPRA 20200123). Clinical trial registration number PACTR202003767867253 and date of trial registration is 18/03/2020. All experiments were performed in accordance with the relevant guidelines and regulations. All participants provided written informed consent.

Blood was collected by venepuncture into acetate citrate dextran (ACD) vacutainer tubes and isolated blood plasma was stored at − 80 °C. Blood samples were collected at enrolment (day 0, prior to antibody administration) and days 1, 7, 28 and 56 post antibody administration. Total lymphocyte counts were performed on an automated XN1000 haematology analyzer (Sysmex). Lymphocytopenia adverse events were graded as per the Division of AIDS (DAIDS) toxicity criteria. According to these criteria, an absolute lymphocyte count is graded as follows: < 0.650 × 10^9^ cells /L (Grade 1: mild), < 0.600 × 10^9^ cells /L (Grade 2: moderate), counts < 0.500 × 10^9^/L (Grade 3: severe) and < 0.350 × 10^9^/L (Grade 4: potentially life-threatening). A woman who experienced gradable lymphocytopenia (referred to as gradable lymphocytopenia women in this analyses) had an absolute lymphocyte count of < 0.650 × 10^9^ cells /L. A total of 8 women experienced at least a Grade 1 lymphocytopenia that resolved within 3–7 days and returned to their original counts at baseline by 7–14 days (Table 1). On the initial day of observation, lymphocytopenia was noted, concomitant with transient non-gradable neutrophilia, as per the Division of AIDS toxicity grading system. Additionally, there was a decrease in eosinophils and monocytes, all of which resolved by day 3 of assessment. Notably, one participant exhibited severe lymphocytopenia and experienced transient grade 1 thrombocytopenia on day 3, which spontaneously resolved within the same day. No other haematological abnormalities were detected during the observation period^17^.

**Table 1:** Assigned active interventions for study participants.

	bnAbs administered	Dose
Gradable lymphocytopenia (n = 8)	CAP256V2LS* + VRC07-523.LS*	10 mg/kg SC + 10 mg/kg SC one dose
	CAP256V2LS	5 mg/kg IV one dose
	CAP256V2LS*	5 mg/kg SC one dose
	CAP256V2LS*	10 mg/kg SC one dose
	CAP256V2LS* + VRC07-523.LS*	10 mg/kg SC + 10 mg/kg SC one dose
	CAP256V2LS*	20 mg/kg SC one dose
	CAP256V2LS*	20 mg/kg SC one dose
	CAP256V2LS	5 mg/kg SC one dose
Non-lymphocytopenia (n = 12)	CAP256V2LS	5 mg/kg IV one dose
	CAP256V2LS	5 mg/kg SC one dose
	CAP256V2LS*	5 mg/kg SC one dose
	CAP256V2LS*	10 mg/kg SC one dose
	CAP256V2LS*	20 mg/kg SC one dose
	CAP256V2LS*	20 mg/kg SC one dose
	CAP256V2LS* + VRC07-523.LS*	10 mg/kg SC + 10 mg/kg SC one dose
	CAP256V2LS*	10 mg/kg SC with one repeat dose at 16 weeks
	CAP256V2LS*	20 mg/kg SC with one repeat dose at 16 weeks
	CAP256V2LS*	10 mg/kg SC with one repeat dose at 16 weeks
	CAP256V2LS*	20 mg/kg SC with one repeat dose at 16 weeks
	CAP256V2LS*	20 mg/kg SC with one repeat dose at 24 weeks

*bnAbs injected with hyaluronidase.

In this sub study, women have been classified as (i) women who have experienced a gradable lymphocytopenia versus (ii) women who experienced a non-lymphocytopenia according to the DAIDS definitions. The woman who received the placebo had a normal lymphocyte count at both pre and post bnAb administration. Therefore, we only included women who were matched in terms of receiving the bnAbs rather than including the placebo women. This was done to preclude confounding related to matching by design. Table 1 shows details on how the women from each of the arms were matched and selected. The comparator groups consisted of twelve women from the same intervention group who did not experience gradable lymphocytopenia and had an absolute lymphocyte count of > 0.650 × 10^9^ cells /L (referred to as non-lymphocytopenia women in this analyses). The safety and PK profiles have been described previously^17^, here we expand on the biological mechanisms underlying the lymphocytopenia.

### IgG subclasses and Ig isotype quantification in plasma

IgG subclasses (IgG1, IgG2, IgG3, IgG4) and isotypes IgM, and IgA were quantified in the plasma using Bio-Plex Pro™ Human Isotyping Panel kit (Bio-Rad, USA) according to the manufacturer’s instructions. Mean Fluorescent Intensity (MFI) were determined by 4-PL logistic regression using the Bioplex Manager 6.0 software (Bio-Rad, Hercules, CA). MFI for the IgG subclasses and isotypes were detected in the linear range of the standard curve.

### Measurement of cytokine concentrations

Concentrations of interleukins (IL)-1β, IL-1Rα, IL-2, IL-4, IL-5, IL-6, IL-7, IL-8, IL-9, IL-10, IL-12p70, IL-13, IL-15, IL-17A, basic fibroblast growth factor (FGF), eotaxin, granulocyte colony-stimulating factor (G-CSF), granulocyte–macrophage colony-stimulating factor (GM–CSF), interferon (IFN)-γ, interferon gamma-induced protein (IP) − 10, monocyte chemotactic protein (MCP) − 1, macrophage inflammatory protein (MIP)–1α, MIP-1β, platelet-derived growth factor (PDGF)-ββ, regulated upon activation normal T cell expressed and presumably secreted (RANTES), tumor necrosis factor (TNF)-α, and vascular endothelial growth factor (VEGF) were measured using Bio-Plex Pro™ Human Cytokine Group I 27-Plex Panel (Bio-Rad, USA) kit. Data were collected using a Bio-Plex Suspension Array Reader (Bio-Rad Laboratories Inc., Hercules, CA) and a 5 PL regression formula was used to calculate sample concentrations from the standard curves. Data were analyzed using Bio-Plex manager software (version 6.1). Cytokine levels on the lower limit of detection (LLOD) of the assay were reported as the mid-point between zero and the lowest concentration measured for that given cytokine. Of the 27 cytokines, 12 cytokines (GM-CSF, IL-10, IL-12p70, IL-15, IL-13, IL-1β, IL-2, IL-5, IL-7, IL-9, PDGF-ββ and VEGF) were below the lower limit of detection (LLOD) for the all the timepoints. To control for inter-plate variability, plasma samples from the same participant were analysed on separate plates.

### Statistical analyses

Baseline characteristics were summarized using medians with interquartile ranges (IQR) for continuous variables and proportions for categorical variables. Additionally, the standard error of the mean (SEM) was utilized to describe the variability of the sample means. Continuous variables were compared between groups using either independent samples t-test or Mann–Whitney U test, depending on the distributional assumptions and normality of the data, and proportions were compared using the Fisher’s exact test. We compared pre- and post-bnAb administration, plasma cytokine concentrations in gradable lymphocytopenia and non-lymphocytopenia groups using the Wilcoxon signed-rank test, due to the non-normality of data. Cytokine concentrations were log transformed to reduce skewness. Two-sided *p*-values less than 0.05 were considered statistically significant. Multiple comparisons adjustment was done using False Discovery Rate (FDR) method of Benjamini and Hochberg with significance level of 5%. Analyses were performed using GraphPad Prism Version 8.4.3 and SAS version 9.4 (SAS Institute Inc., Cary, NC, US).

## Results

### Study participants

The baseline characteristics of the study participants are summarized in Table 2. The median age was 25 years (IQR 22.25–26.75) for gradable lymphocytopenia women and 21 years (IQR 20–25) for non-lymphocytopenia women. Of the eight lymphocytopenia women, two developed grade 1, two grade 2, two grade 3 and two grade 4 lymphocytopenia. In addition, the body mass index (BMI) was significantly lower in gradable lymphocytopenia women with a mean of 22.27 (SD = 4.03)compared to non-lymphocytopenia women who had a mean of 28.58 (SD = 6.22) (*p* = 0.021). No significant differences were observed between gradable lymphocytopenia and non-lymphocytopenia women for education, weight, vaginal and anal sex acts in the last 30 days and condom use. Women in both groups had stable partners. Depo-provera was the most common contraceptive for both groups (62.50% and 66.66% respectively). In gradable lymphocytopenia women, 62.50% had intermediate bacterial vaginosis (BV) and 25.00% and 50.00% of women had BV and intermediate BV respectively among non-lymphocytopenia women. Furthermore, no women had any sexually transmitted infections (STIs).

**Table 2:** Baseline characteristics of gradable lymphocytopenia and non-lymphocytopenia women.

	Gradable lymphocytopenia (n = 8)	Non-lymphocytopenia (n = 12)	*p*-value
Age (years) median (IQR)	25.0 (22.25–26.75)	21.00 (20.00–25.00)	ns
Highest education level:			ns
Primary n (%)	1 (12.50)	0	
Secondary n (%)	5 (62.50)	11 (91.6)	
Tertiary n (%)	2 (25.00)	1 (8.33)	
Weight (kg) mean (SD)	57.95 (11.10)	66.57 (14.66)	ns
Height (m) mean (SD)	1.61 (0.03)	1.53 (0.03)	< 0.001
BMI mean (SD)	22.27 (4.03)	28.58 (6.22)	0.021
Relationship status:			
Stable partner n (%)	8 (100)	12 (100)	
Current partner ever tested positive:			ns
Yes	0	0	
No n (%)	8 (100)	12 (100)	
Age (years) at sexual debut mean (SD)	19.63 (2.26)	17.50 (1.31)	0.016
Vaginal sex in the last 30 days:			
Yes n (%)	4 (50.00)	9 (75.00)	
No n (%)	4 (50.00)	3 (25.00)	
Anal sex in the last 30 days:			ns
Yes n (%)	0	0	
No n (%)	8 (100)	12 (100)	
Condom use during Vaginal or anal sex:			ns
Sometimes	6 (75.00)	9 (75.00)	
Always	2 (25.00)	3 (25.00)	
Diagnosed or treated for STI in the last 30 days:			ns
Yes n (%)	0	0	
No n (%)	8 (100)	12 (100)	
Current contraceptive methods:			ns
Depo Provera n (%)	5 (62.50)	8 (66.66)	
Nuristerate n (%)	3 (37.50)	4 (33.33)	
Ever taken a non-prescription drug:			ns
Yes n (%)	0	0	
No n (%)	8 (100)	12 (100)	
Nugent score:			ns
No BV n (%)	3 (37.50)	3 (25.00)	
Intermediate BV n (%)	5 (62.50)	6 (50.00)	
BV n (%)	0	3 (25.00)	
STIs:			
*Trichomoniasis*	0	0	
*Neisseria gonorrhoea*	0	0	
*Chlamydia trachomatis*	0	0	

*IQR-interquartile range, Ab-antibody, BV-bacterial vaginosis, STIs-sexually transmitted infection, *p*-value < 0.05 is significant, ns-not significant. Means were compared using the independent samples t-test while medians were compared using the Mann–Whitney U test. Proportions were compared using the Fisher’s exact test.

### Kinetics of blood lymphocyte count within gradable lymphocytopenia and non-lymphocytopenia women

**Figure 1:**
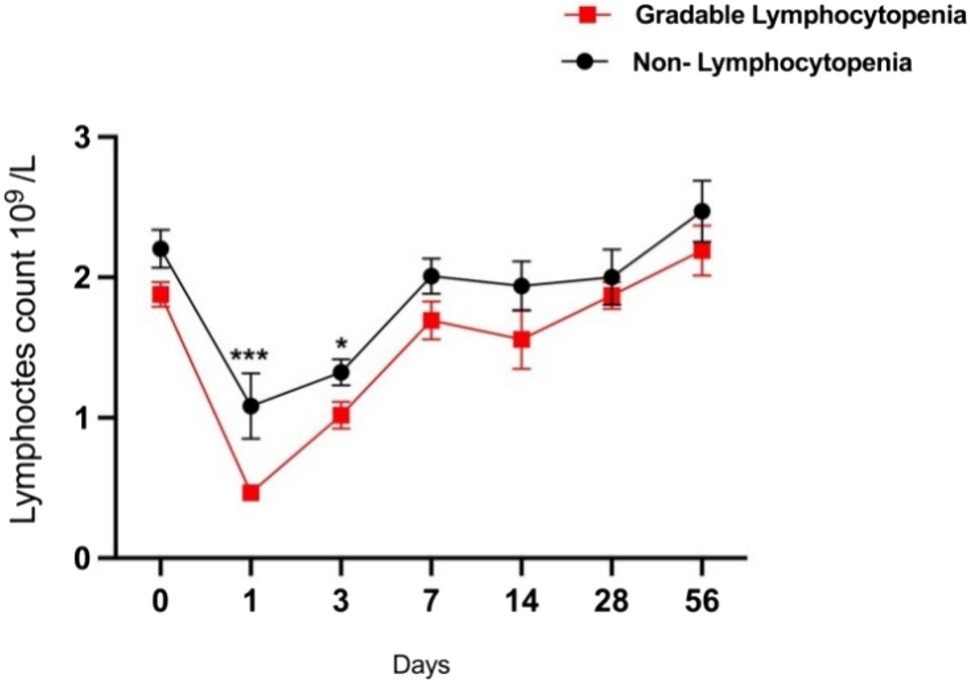
Kinetic analysis of blood lymphocyte counts in gradable lymphocytopenia women (n = 8) and non-lymphocytopenia women (n = 12) from Day 0 to Day 56. Data shows mean and standard error of mean (SEM) of lymphocyte count over time from day 0 prior to bnAbs administration to 72 days after bnAbs administration. The unpaired t test was used to compare the means between the two groups. *p* value < 0.05 is statistically significant. Asterix (*) denotes *p* < 0.05 and (***) *p* < 0.005.

Gradable lymphopenia was observed in eight women after bnAbs administration. We analysed changes in kinetics of lymphocyte count in the blood of women before and after bnAb administration (Fig. 1). Overall, there was a drop in lymphocytes, however a significant difference in the lymphocyte count was observed in gradable lymphocytopenia women after bnAb administration at day 1 compared to non-lymphocytopenia women at Days 1 and 3 (*p* < 0.005 and *p* < 0.050 respectively). From day 3 of bnAb administration, the lymphocyte counts gradually increased in the gradable lymphocytopenia women. No significant differences in the lymphocyte counts between gradable lymphocytopenia and non-lymphocytopenia women were observed from day 7 to day 56.

### Plasma IgG subclasses and IgM were significantly lower in gradable lymphocytopenia women compared to non-lymphocytopenia women pre-and post-CAP256V2LS administration

**Figure 2:**
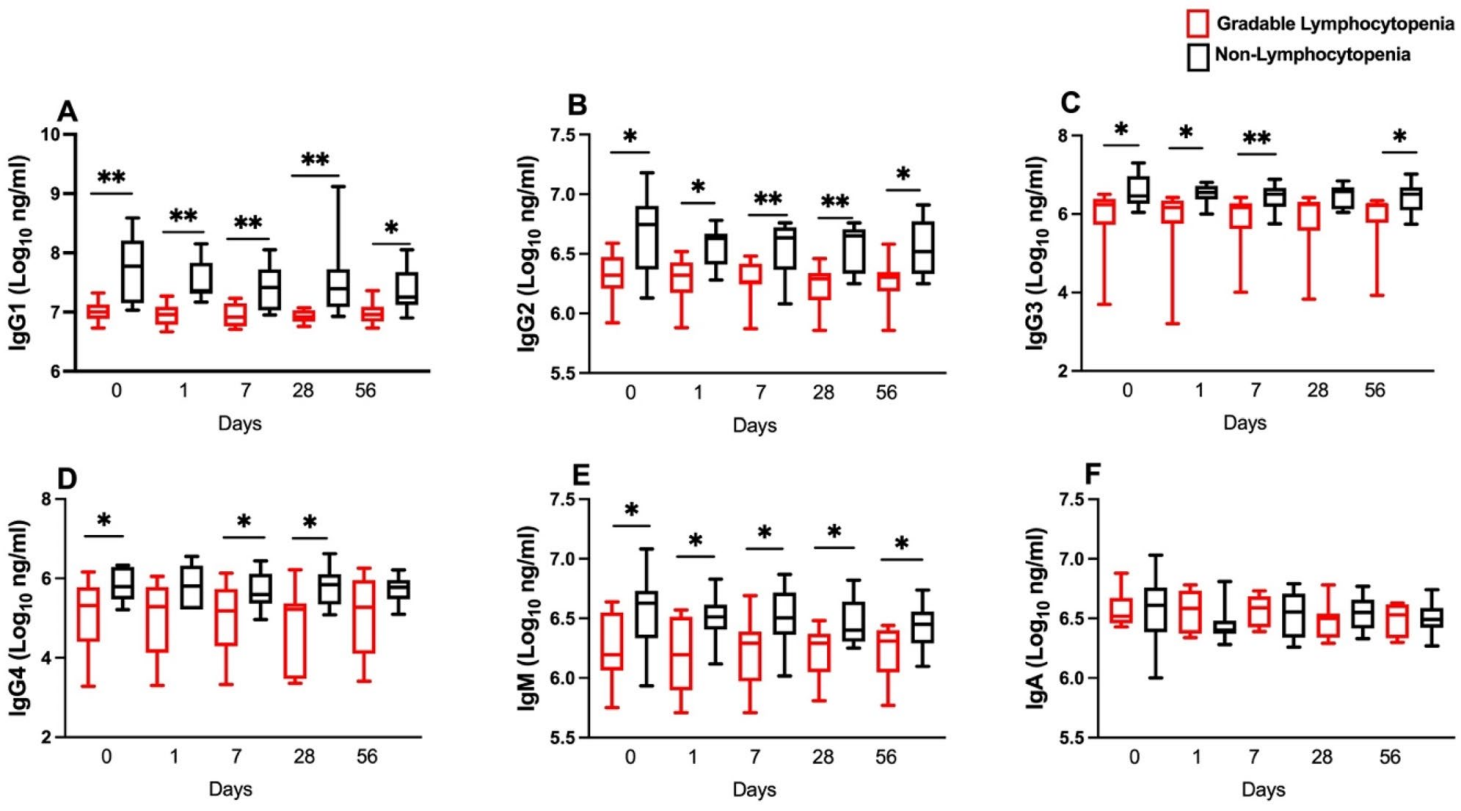
Plasma IgG subclasses: (**A**) IgG1, (**B**) IgG2, (**C**) IgG3, (**D**) IgG4 and isotypes: (**E**) IgM and (**F**) IgA (Log_10_ ng/ml) between women with gradable lymphocytopenia (n = 8) and non-lymphocytopenia (n = 12) from Day 0 to Day 56. The Mann–Whitney U test was used to compare the medians between the two groups at different timepoints. *p* value < 0.05 is considered statistically significant. Asterix (*) denotes *p* < 0.05 and (**) *p* < 0.01.

We determined plasma IgG subclasses and isotypes in gradable lymphocytopenia women compared to the non-lymphocytopenia women (Fig. 2). All IgG subclasses (IgG1, IgG2 and IgG3 and IgG4) and isotype IgM were significantly lower (*p* < 0.05) at enrolment (Day 0) among gradable lymphocytopenia women compared to non-lymphocytopenia women (Fig. 2A–E). Furthermore, the IgG subclasses and IgM isotypes remained significantly lower among the gradable lymphocytopenia women on days 1, 7, 28 and 56 post antibody administration relative to non-lymphocytopenia women. In contrast, IgA was similar.

### Lower TNF-α and higher MIP-1β levels among gradable lymphocytopenia women

The concentrations of 27 cytokines were measured in the plasma samples within the gradable lymphocytopenia and non-lymphocytopenia women at different timepoints. Of the 27 cytokines, 12 cytokines (GM-CSF, IL-10, IL-12p70, IL-15, IL-13 IL-1β, IL-2, IL-5, IL-7, IL-9, PDGF-ββ and VEGF) were below the lower limit of detection (LLOD) for all the timepoints. However, 15 of the 27 cytokines (Basic FGF, eotaxin, G-CSF, IFN-γ, IL-17A, IL-1RA, IL-4, IL-6, IL-8, IP-10, MCP-1, MIP-1α, MIP-1β, RANTES, TNF-α) were significantly elevated at day 1 post antibody administration compared to baseline in gradable lymphocytopenia women (Table 3). This statistical association remained significant after multiple comparisons adjustment. Within the non-lymphocytopenia women, 6 cytokines (G-CSF, IL-1RA, IL-6, IL-8, IP-10 and MCP-1) were significantly higher at day 1 compared to day 0. G-CSF, IL-6 and MCP-1 remained significant after multiple comparisons adjustment.

**Table 3:** Comparison of cytokine expression between day 0 and day 1 within gradable lymphocytopenia and non-lymphocytopenia women.

Plasma Cytokines	Plasma cytokine concentration (Log10 pg/ml) Median (IQR)		*p*-value	FDR	Plasma cytokine concentration (Log10 pg/ml) Median (IQR)		*p*-value	FDR
	Gradable lymphocytopenia				Non-lymphocytopenia			
	Day 0	Day1			Day 0	Day 1		
Basic FGF	0.934 (0.764–1.107)	1.284 (1.229–1.354)	0.009	0.029	1.158 (1.031–1.219)	1.311 (1.069–1.519)	0.176	0.209
Eotaxin	0.887 (0.786–0.921)	1.268 (1.210–1.393)	0.001	0.006	0.916 (0.769–1.008)	1.097 (0.936–1.515)	0.063	0.093
G-CSF	1.480 (1.323–1.622)	2.315 (2.197–2.410)	0.016	0.030	1.539 (1.501–1.692)	2.013 (1.878–2.291)	0.011	0.030
IFN-γ	0.268 (− 0.062–0.441)	1.131(0.996–1.222)	0.002	0.009	0.630 (0.210–0.758)	0.85 (0.721–1.758)	0.078	0.010
IL-17A*	− 0.111(− 0.111–0.212)	0.492 (0.259–0.641)	0.039	0.062	–0.347 (− 0.347–0.242)	0.093 (–0.347–0.887)	0.312	0.334
IL-1Ra	2.256 (2.151– 2.325)	3.046 (2.723 –3.250)	0.001	0.006	2.321 (2.229–2.359)	2.664 (2.460–3.161)	0.035	0.059
IL-4	0.173 (0.140–0.325)	0.438 (0.368–0.507)	0.007	0.028	0.086 (− 0.049–0.195)	0.152 (–0.046–0.426)	0.207	0.237
IL-6*	− 0.585 (− 0.585– − 0.585)	1.300 (1.113–1.496)	0.006	0.028	–1.125 (–1.125– − 0.092)	0.702 (0.506–1.208)	0.016	0.030
IL-8	0.124 (0.124–0.124)	1.103 (0.893–1.337)	0.016	0.030	0.347 (0.147–0.477)	0.713 (0.506–0.935)	0.046	0.070
IP-10	1.878 (1.751–1.903)	3.743 (3.288–3.905)	< 0.001	0.002	1.931 (1.887–2.174)	3.576 (2.937–3.961)	0.031	0.055
MCP-1	0.835 (0.708–0.931)	2.240 (2.018–2.552)	< 0.001	0.002	0.877 (0.781–0.931)	1.547 (1.261–2.570)	0.013	0.030
MIP-1α	− 0.289 (–1.260– − 0.165)	0.919 (0.769–1.080)	0.016	0.030	− 0.111 (–0.534– − 0.014)	0.439 (0.281–0.852)	0.078	0.100
MIP-1β	1.867 (1.852–1.890)	2.150 (2.043–2.328)	0.001	0.006	1.726 (1.708–1.752)	1.803 (1.745–2.036)	0.287	0.317
RANTES	3.081 (2.959–3.162)	3.236 (3.173–3.385)	0.016	0.030	3.014 (2.868–3.182)	3.004 (2.915–3.324)	0.937	0.938
TNF-α	1.295 (1.178–1.359)	1.559 (1.489–1.671)	0.002	0.009	1.413 (1.377–1.453)	1.57 (1.428–1.737)	0.154	0.190

*Cytokine levels below LLOD were reported as the mid-point between zero and the lowest concentration measured for that given cytokine. Medians at Day 0 and Day 1 were compared using the Wilcoxon signed- rank test. FDR-False discovery rate, *p* < 0.05 is statistically significant.

**Figure 3:**
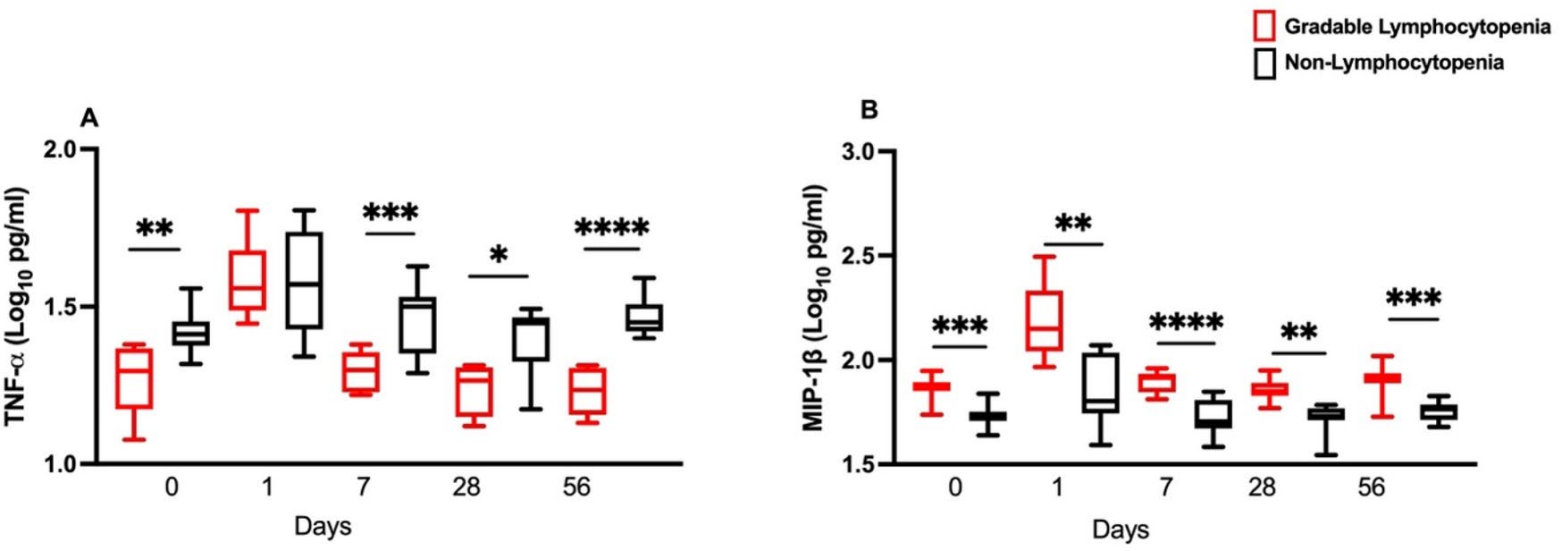
(**A**) TNF-α and (**B**) MIP-1β (Log_10_ pg/ml) in the blood for gradable lymphocytopenia (n = 8) compared to the non-lymphocytopenia women (n = 12) at several timepoints pre- and post- bnAb administration. The Mann–Whitney U test was used to compare the medians between the two groups. *p* value < 0.05 is considered statistically significant. Asterix (*) denotes *p* < 0.05, (**) *p* < 0.01, (***) *p* < 0.001and (****) *p* < 0.0001.

When the gradable lymphocytopenia women were compared to the non-lymphocytopenia women, pro-inflammatory cytokine TNF-α was significantly lower in gradable lymphocytopenia women compared to non- lymphocytopenia women (*p* < 0.05) on days 0, 7, 28, and 56, but not on day 1 where no difference could be seen. (Fig. 3A). In contrast, chemokine MIP-1β was significantly higher (*p* < 0.05) in gradable lymphocytopenia women relative to non-lymphocytopenia women at all timepoints (Fig. 3B).

## Discussion

In this trial, the development of lymphocytopenia was a physiological event associated with some inflammatory markers, however this phenomenon had no apparent clinical effects and resolved within days.

In this study, a universal decrease in lymphocytes after bnAb administration was observed. However, of importance, 8 women developed a gradable lymphocytopenia. These women had concomitant lower plasma IgG subclasses and IgM compared to non-lymphocytopenia women prior to and until day 56 post bnAbs administration. These data suggest that in addition to a lower baseline lymphocyte count, lower concentrations of Igs may be predictive of the onset of gradable lymphocytopenia during passive immunotherapy. The exact biological mechanism of how the lower Ig concentrations predispose to the development of lymphocytopenia during passive immunotherapy using bnAbs remains elusive. In support of these findings, a COVID-19 study showed a significant association between IgG deficiency at baseline and lower CD4^+^/CD8^+^ T cells and CD19^+^ B cells^13^. There is scant data on the deficiency of IgG subclasses and isotypes IgM and IgA either alone or in combination and the predilection for onward lymphocytopenia. Whether this Ig deficiency augments the lymphocytopenia when an intervention is given, remains undetermined. It should be noted that in addition, the gradable lymphocytopenia women did not satisfy the diagnostic criteria for hypogammaglobulinemia which is defined as decreased levels of IgG, IgA and/or IgM, 2 standard deviations below the mean for age^18,19^. However, despite this, it does not necessarily preclude them from being at risk for developing lymphocytopenia. Although the gradable lymphocytopenia women had significantly lower BMI compared to non-lymphocytopenia women, their BMIs were within the normal range. Previous studies have shown that a lower BMI is a significant risk factor for developing lymphocytopenia in several diseases such as TB, HIV, and cancer^20–22^. Our finding of gradable lymphocytopenia following bnAbs in women with lower BMI needs further investigation to establish if BMI may be a predictor for the development of lymphocytopenia.

Several studies confirm the critical role of increased inflammatory cytokines during the onset of lymphocytopenia^23^. The action of cytokines, chemokine and growth factors can synergise to inhibit T-cell proliferation^24^. Within 24 h of CAP256-V2LS administration, there was a significant increase in several chemokines, adaptive, growth factors and pro-inflammatory cytokines in lymphocytopenia women. Pre-existing levels of cytokines were significantly modified post antibody administration. Circulating cytokines can affect the development of lymphocytopenia through several mechanisms. Firstly, the pro-inflammatory cytokine, TNF-α can enhance T-lymphocyte apoptosis leading to T cell depletion^25–27^. We observed a temporal increase in several cytokines after bnAb administration that coincided with gradable lymphocytopenia. However, e observed significantly lower TNF-α in the gradable lymphocytopenia women when compared to non-lymphocytopenia women at most timepoints except for day 1 only post-bnAbs where no difference was seen. Previous studies have demonstrated, that TNF-α secreted by B cells, plays an autocrine role in B cell proliferation^28,29^. Therefore, among gradable lymphocytopenia women, lower baseline TNF-α potentially leads to lower numbers of B cells and potentially immature B cells resulting in lower Ig levels. Furthermore, TNF ligand superfamily (TNFSF) signal apoptosis proliferation-inducing ligand (APRIL) and B cell-activating factor (BAFF) of the TNF family play a key role in B cell development^30^. Therefore, it is biologically plausible that a low Ig profile among gradable lymphocytopenia women may be attributed to significantly decreased TNF-α levels. Secondly, IFN-γ regulates lymphocyte recirculation and induces transient blood lymphocytopenia^31^. Thirdly, cytokines can regulate adhesion molecules such as intercellular adhesion molecules (ICAM)-1 and vascular cell adhesion molecule (VCAM)-1 and redirect or mobilize T cells in circulation to other sites resulting in low circulating lymphocytes^32^. Both MIP-1α and MIP-1β can trigger chemotaxis and adhesion of T cells to the adhesion molecules by activation causing temporary margination of the lymphocytes^33,34^. MIP-1β was significantly and consistently elevated in gradable lymphocytopenia women compared to non-lymphocytopenia women at all timepoints. MIP-1β, in addition to its chemotactic role, triggers the adhesion of T cells to the VCAM-1, a mechanism that may be increasing their “stickiness” to the vascular endothelium, augmenting T cell margination^33^, another mechanism contributing to the transient and acute lymphocytopenia.

In this sub study, even though the development of the lymphocytopenia was not clinically significant and transient, understanding its kinetics and duration is important. The transient lymphocytopenia indicates a need for intensive safety monitoring of cell counts following administration of bnAbs. This monitoring may help to establish if there are any immunomodulatory effects of the bnAbs. It may also help define threshold levels and drops in lymphocyte counts post bnAb administration. This may aid in optimizing dosing schedules or route of administration. The specific mechanisms behind transient lymphocytopenia needs to be further investigated. Potential mechanisms include redistribution where bnAbs might cause lymphocytes to migrate from the bloodstream to lymphoid tissues or other tissues, manifesting as a blood lymphocytopenia^35,36^. Indeed some monoclonal antibodies used in cancer therapies for example might directly or indirectly induce lymphocyte apoptosis or inhibit lymphocyte proliferation, either a deliberate effect or an off-target and unintended consequence^37^. The formation of immune complexes elicited by monoclonal antibodies might engage immune cells differently, leading to temporary reductions in circulating lymphocytes^38,39^. Understanding these mechanisms is crucial for developing strategies to manage and mitigate any potential adverse effects that may undermine bnAbs as passive immunoprophylaxis for HIV.

The present study has several limitations. There was a limited sample size of participants who experienced gradable lymphocytopenia. Another limitation was the lack of contemporaneous peripheral blood mononuclear cells (PBMCs) from these women when they developed the lymphocytopenia to show potential mechanisms between the cellular phenotypes, immune activation, and the corresponding cytokine profiles. A further observation is that despite the non-lymphocytopenia women experiencing a decline in the total lymphocytes post bnAbs, their decline did not satisfy the minimum for gradable lymphocytopenia as per the DAIDS definition utilised in clinical trials. The non-lymphocytopenia women had three biological features that distinguished them from the gradable lymphocytopenia women, significantly higher Ig subclasses/IgM, BMI and a modestly higher lymphocyte count. The sample size precluded the matching of participants based on BMI alone. However, gradable lymphocytopenia and non-lymphocytopenia women were matched on the arm of the study depending on whether women received CAP256V2LS alone or in combination with VRC07-523LS. Perhaps there is a biological threshold of Ig subclasses/IgM combined with BMI that predispose towards gradable lymphocytopenia in the context of passive immunization. The advantage of this study is that the clinical parameters for assessing the safety of the bnAbs was conducted intensively for the first few days of evaluation. This aided in the prompt diagnosis of gradable lymphocytopenia. Consequently, the baseline and subsequent follow-up samples and data enabled us to better define the profiles of the immunoglobulins and cytokines as putative prognostic markers. The development of prognostic markers will help identify participants at high risk for developing lymphocytopenia prior to drug administration. Ultimately, prognostic markers are useful in clinical decision-making and allow timely interventions with the therapies that might improve study outcomes.

In conclusion, our study provides a critical context for the development of gradable lymphocytopenia with significantly lower baseline Ig profiles in women receiving bnAbs as passive immuno-prophylaxis. We do not know if an increase of lymphocytopenia severity will occur with repeat administration of CAP256V2LS.These findings suggest that in addition to lower baseline lymphocyte counts, reduced baseline Igs found during testing of HIV monoclonal antibodies may be used as a surrogate for identifying participants in clinical trials at risk of developing lymphocytopenia. Concordant with other monoclonal antibody studies, there were increased cytokines in women with gradable lymphocytopenia^40–42^. Of note, in particular, two cytokines TNF-α (except for day 1) and MIP-1β, maintained the same kinetics or pattern at both pre and post bnAbs for gradable lymphocytopenia compared to non-lymphocytopenia women up to day 56. Together, cytokines and Igs play a critical role in the regulation of peripheral blood cell trafficking, immune cell activation and inflammation. Future studies are needed to fully understand the interplay between these biological mediators in the development of lymphocytopenia which may be of clinical relevance when evaluating bnAbs for HIV prevention in at-risk populations.

## Data Availability

The datasets used and/or analysed during the current study available from the corresponding author on reasonable request.
